# Kinetic Studies on the Catalytic Degradation of Rhodamine B by Hydrogen Peroxide: Effect of Surfactant Coated and Non-Coated Iron (III) Oxide Nanoparticles

**DOI:** 10.3390/polym12102246

**Published:** 2020-09-29

**Authors:** Mohd Shaban Ansari, Kashif Raees, Moonis Ali Khan, M.Z.A. Rafiquee, Marta Otero

**Affiliations:** 1 Department of Applied Chemistry, Zakir Hussain College of Engineering and Technology, Aligarh Muslim University, Aligarh 202002, UP, India; shabanansari126@gmail.com (M.S.A.); raeeskashif@gmail.com (K.R.); 2 Chemistry Department, College of Science, King Saud University, Riyadh 11451, Saudi Arabia; mokhan@ksu.edu.sa; 3 CESAM—Centre for Environmental and Marine Studies, Department of Environment and Planning, University of Aveiro, Campus de Santiago, 3810-193 Aveiro, Portugal

**Keywords:** magnetite, co-precipitation method, Rhodamine B, sodium dodecyl sulfate, wastewater treatment

## Abstract

Iron (III) oxide (Fe_3_O_4_) and sodium dodecyl sulfate (SDS) coated iron (III) oxide (SDS@Fe_3_O_4_) nanoparticles (NPs) were synthesized by the co-precipitation method for application in the catalytic degradation of Rhodamine B (RB) dye. The synthesized NPs were characterized using X-ray diffractometer (XRD), vibrating sample magnetometer (VSM), scanning electron microscopy (SEM), transmission electron microscopy (TEM), and Fourier transform infra-red (FT-IR) spectroscopy techniques and tested in the removal of RB. A kinetic study on RB degradation by hydrogen peroxide (H_2_O_2_) was carried out and the influence of Fe_3_O_4_ and SDS@Fe_3_O_4_ magnetic NPs on the degradation rate was assessed. The activity of magnetic NPs, viz. Fe_3_O_4_ and SDS@Fe_3_O_4_, in the degradation of RB was spectrophotometrically studied and found effective in the removal of RB dye from water. The rate of RB degradation was found linearly dependent upon H_2_O_2_ concentration and within 5.0 × 10^−2^ to 4.0 × 10^−1^ M H_2_O_2_, the observed pseudo-first-order kinetic rates (k_obs_, s^−1^) for the degradation of RB (10 mg L^−1^) at pH 3 and temperature 25 ± 2 °C were between 0.4 and 1.7 × 10^4^ s^−1^, while in presence of 0.1% *w*/*v* Fe_3_O_4_ or SDS@Fe_3_O_4_ NPs, k_obs_ were between 1.3 and 2.8 × 10^4^ s^−1^ and between 2.6 and 4.8 × 10^4^ s^−1^, respectively. Furthermore, in presence of Fe_3_O_4_ or SDS@Fe_3_O_4_, k_obs_ increased with NPs dosage and showed a peaked pH behavior with a maximum at pH 3. The magnitude of thermodynamic parameters E_a_ and ΔH for RB degradation in presence of SDS@Fe_3_O_4_ were 15.63 kJ mol^−1^ and 13.01 kJ mol^−1^, respectively, lowest among the used catalysts, confirming its effectiveness during degradation. Furthermore, SDS in the presence of Fe_3_O_4_ NPs and H_2_O_2_ remarkably enhanced the rate of RB degradation.

## 1. Introduction

Mushrooming industrialization and urbanization are primarily responsible for deteriorating the surface and sub-surface water quality, causing hazardous effects on both aquatic organisms and human health. Among water contaminants, dyes and pigments, which are widely discharged from textile, pharmaceutical, paint, rubber, cosmetic, and confectionary industries effluents [1,2], produce unwanted color to water bodies, resulting in intoxication of ecosystems. Rhodamine B (RB) is a synthetic cationic dye, containing a multi-ring aromatic xanthene core planar structure [3]. It is widely used for dyeing and printing applications [4]. The carcinogenic, mutagenic, and toxic effects of RB have been well reported [5,6,7], evidencing the need of RB contaminated effluents treatment prior to their discharge. Various treatment methodologies, such as reverse osmosis, ion-exchange, precipitation, adsorption, ozone treatment, catalytic reduction, biodegradation, ultrasonic decomposition, coagulation, electrocoagulation, chemical oxidation, and nano-filtration, have been used for the removal of RB and other dyes from water [8,9,10]. However, high-cost, long process duration, large energy consumption, regeneration difficulties, and pollutants transfer from one phase to another are the major demerits of the aforementioned processes. Thus, advanced oxidation processes (AOPs) are considered comparatively advantageous since they possess favorable decolorizing ability for reactive dyes [11]. Fenton reaction, which is one of the most effective AOPs, has attracted widespread attention. It is operated at acidic pH in the presence of hydrogen peroxide (H_2_O_2_) and ferrous ions while yielding hydroxyl radical with powerful oxidation capacity leading to complete decomposition of organic dyes, thus, converting them into non-toxic lower molecular weight products [12]. In this sense, the Fe^2+^-H_2_O_2_ Fenton system has been widely used for the oxidative removal of RB from water [11,12,13].

Recently, iron (III) oxide (Fe_3_O_4_) nanoparticles (NPs) have been used for removing various dyes and heavy metals from water [14,15,16]. These NPs are inert, economical, possess unique magnetic properties, and can be easily separated from reaction medium through an external magnetic field [17,18,19]. Additionally, Fe_3_O_4_ magnetic NPs exhibit a high surface area, depending on the particle size, and show the ability for surface modification. Furthermore, the interaction of Fe_3_O_4_ NPs with H_2_O_2_ generates hydroxyl and peroxyl radicals, which are able to undergo the oxidative degradation of organic pollutants [20,21,22]. However, bare Fe_3_O_4_ NPs suffer some shortcomings such as agglomeration, limited adsorption ability, and limited working pH range. Coating of Fe_3_O_4_ NPs with surfactants, polymers, silica, starch, polyelectrolytes, etc., render an enhancement in their surface properties and chemical stability, making them suitable for industrial wastewater treatment and catalytic applications [23,24,25,26,27,28,29]. Among surfactants used for coating, the anionic sodium dodecyl sulfate (SDS) is known to enhance the ability of NPs to remove pollutants from wastewater, which has been related with the binding and chelating efficiency of its functional groups [30]. Although no studies were found on the specific case of RB, Fe_3_O_4_ NPs modified with SDS have been successfully used for the adsorptive removal of several dyes from water, including tolonium chloride [31], Basic Blue 41 [32], or Brilliant Green [33].

The present work was undertaken with the aim of developing an efficient, eco-friendly, and economical treatment for the removal of cationic dyes from water. For this purpose, Fe_3_O_4_ NPs were synthesized, coated with SDS, and tested as catalyst for the degradation of RB under the presence of H_2_O_2_. The synthesized Fe_3_O_4_ and SDS-coated Fe_3_O_4_ (SDS@Fe_3_O_4_) NPs were thoroughly characterized through XRD, VSM, SEM, TEM, and FT-IR techniques. The main novelty of this work was the comparative study of the dye degradation by H_2_O_2_ under three different situations, namely, in absence of ferrous NPs, in the presence of Fe_3_O_4_ NPs, and in the presence of SDS@Fe_3_O_4_ NPs. Kinetic experiments were carried out to explore the influence of these catalysts dosages, H_2_O_2_, SDS, and solution pH on the RB degradation rate. 

## 2. Materials and Methods 

### 2.1. Chemicals and Reagents

Rhodamine B (RB: AR grade 80%; CDH, New Delhi, India), hydrochloric acid (HCl: AR grade 36%; Fisher Scientific, Mumbai, India), hydrogen peroxide (H_2_O_2_: 35% *v*/*v*, Merck, Mumbai, India), sodium dodecyl sulphate (SDS: 99%; CDH, New Delhi, India), Ammonia solution (NH_4_OH: 25% with purity index 99%, Thermo Fisher Scientific, Mumbai, India), ferrous chloride dihydrate (FeCl_2_. 2 H_2_O: 99%; CDH, New Delhi), ferric Chloride (FeCl_3_: 97.0%; CDH, New Delhi, India), and sodium hydroxide pellets (NaOH: 97%, Merck, Mumbai, India) were used as supplied. All the other reagents used during the experimental work were of reagent grade. All the solutions were prepared in deionized (DI) water. The stock solutions of NaOH (1.0 M) and SDS (1.0 × 10^−2^ M) were prepared in DI water. The stock solution (500 mgL^−1^) of dye was prepared by dissolving 50 mg RB in 100 mL DI water. Likewise, 250 mL stock solution of H_2_O_2_ was prepared by dissolving 25 mL of H_2_O_2_ in DI water. The stock solution of HCl (0.1 M) was prepared in 100 mL DI water.

### 2.2. Synthesis and Surfactant Coating of Fe_3_O_4_ Magnetic NPs 

Magnetic nanoparticles were synthesized by adopting the co-precipitation method as described in the literature [34]. Briefly, Fe_3_O_4_ NPs were synthesized by mixing 20.0 g of FeCl_3_ (0.4 M) and 10.0 g of FeCl_2_.2H_2_O (0.2 M) into a 1.0 L conical flask. These iron salts were dissolved in 300 mL DI water. The mixture was purged with N_2_ gas and stirred for about an hour. Then, liquor ammonia (25%) was added drop-wise in the flask. The pH of the solution in flask was further increased to ~10 by adding 2.0 M of NaOH solution. The temperature of the solution was then raised to 70 °C with stirring and purging of N_2_ gas for 5 h. Black precipitate was formed in the flask. It was filtered, washed with acetone, and thereafter with DI water to a neutral pH value. The precipitate was then dried at 70 °C in a vacuum oven. The synthesis of Fe_3_O_4_ NPs can be given by the following reaction:

$${\;\mathrm{Fe}}^{2+}+2{\mathrm{Fe}}^{3+}+8{\mathrm{OH}}^{-}\to {\;\mathrm{Fe}}_{3}O_{4}+4H_{2}O$$

To prepare the SDS-coated Fe_3_O_4_ NPs, FeCl_3_.6H_2_O (20 g, 0.40 M), FeCl_2_.4H_2_O (10 g, 0.20 M), and SDS (8.64 g, 0.10 M) were taken into the conical flask of 1.0 L capacity containing 300 mL DI water. The overhead stirrer was used to mix the reactants properly. The solution was stirred vigorously for 45 min under the N_2_ gas atmosphere. Then, 200 mL of 25% ammonium hydroxide solution was added drop-wise into the above solution until the pH of the resulting solution reached 9–11. The pH of the reaction medium was further raised to 14 by adding 2.0 M NaOH solution drop-wise. The mixture was then stirred vigorously under N_2_ gas purging for 5 h. The black precipitate that formed was filtered and washed with acetone and DI water until the pH came to a neutral value.

### 2.3. Characterization

The crystallinity and phase composition of Fe_3_O_4_ and SDS@Fe_3_O_4_ NPs were studied by X-ray diffraction (XRD: MiniFlex II, Rigaku, Tokyo, Japan) analysis equipped with a Cu K_α_ radiation source (with λ = 1.5406 nm). The surface functionalities present over Fe_3_O_4_ and SDS@Fe_3_O_4_ NPs surface were determined by Fourier infra-red spectrometer (FT-IR: Nicolet iS50, Thermo Fisher Scientific, Madison, WI, USA). The surface morphology and particle size were analyzed by scanning electron microscopy (SEM: JSM-5600LV, JEOL, Tokyo, Japan) and transmission electron microscopy (TEM: CM120, Philips, Amsterdam, The Netherlands). The magnetic properties of Fe_3_O_4_ and SDS@Fe_3_O_4_ NPs were determined using a vibrating sample magnetometer (VSM: 7307, Lakeshore, Westerville, OH, USA).

### 2.4. Degradation Kinetic Experiments

A Genesys 10S UV–visible spectrophotometer (Thermo Fisher Scientific, Madison, WI, USA) was used to monitor the change in the absorbance intensity of RB during its degradation under the varying reaction conditions. The spectrophotometer was provided with multiple cell holders in which a 3.0 mL quartz cuvette with a path length of 10 mm was used to measure absorbance. All the kinetic experiments were performed at a constant temperature of 25.0 ± 0.2 °C by using a thermostatic water-bath. A 0.1% *w*/*v* of magnetic Fe_3_O_4_ NPs was put together with RB solution with an initial concentration of 10 mg L^−1^ into a three necked round bottom flask of 100 mL capacity. Solution pH was adjusted by adding hydrochloric acid or sodium hydroxide solution and monitored by using a pH meter. The reaction vessel containing RB solution and magnetic Fe_3_O_4_ NPs was kept in the water-bath to equilibrate with the required temperature. The reaction was started with the addition of 5.0 × 10^−2^ to 4.0 × 10^−1^ M H_2_O_2_ and zero time was taken when the half of the amount of H_2_O_2_ was added. The concentration of RB was spectrophotometrically analyzed at its maximum absorbance wavelength (λ_max_: 554 nm) at constant time intervals. All the kinetic experiments were carried out under pseudo-first-order conditions in which H_2_O_2_ was kept in excess over RB. The progress of the reaction gradually resulted in the decrease of RB concentration and the values of the pseudo-first-order rate constants were obtained from the slopes of the plots of ln (absorbance) versus time. Each kinetic run was carried out in triplicate to check their repeatability and the rate constant was observed to be within the error limits of ~5%.

## 3. Results and Discussion

### 3.1. Characterization of Fe_3_O_4_ and SDS@Fe_3_O_4_ NPs

#### 3.1.1. X-ray Diffraction (XRD)

**Figure 1:**
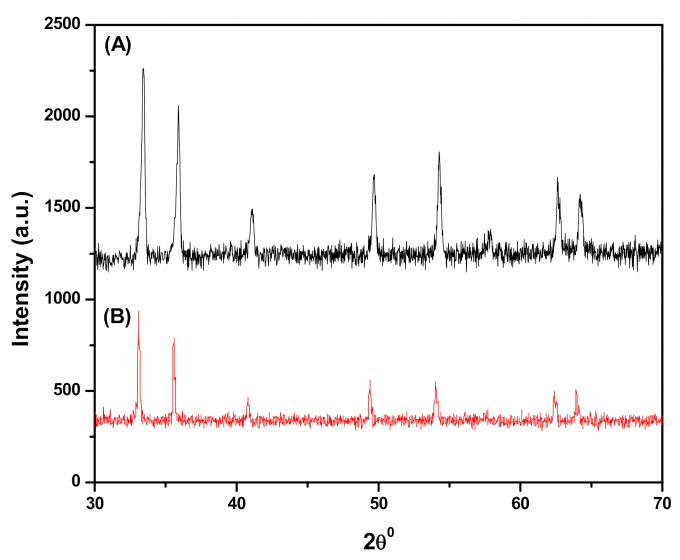
X-ray diffraction patterns for synthesized iron (III) oxide (Fe_3_O_4_) (**A**) and sodium dodecyl sulfate (SDS) coated iron (III) oxide (SDS@Fe_3_O_4_) (**B**).

Figure 1A shows the XRD patterns obtained for the synthesized Fe_3_O_4_ NPs and it confirms the nanocrystal structure and phase purity of Fe_3_O_4_ NPs. The diffraction peaks appeared at 2θ = 30.26°, 35.5°, 43.12°, 53.74°, 57.10°, and 62.92° corresponding to planes (220), (311), (400), (422), (511), and (440), respectively [35], consistent with standard magnetite database (JCPDS-19-0629), indicating a highly crystalline nature of Fe_3_O_4_ NPs. Figure 1B shows the XRD patterns for SDS@Fe_3_O_4_ NPs with reduced peak intensity due to the SDS coating over Fe_3_O_4_ surface. This confirms crystalline-to-amorphous transition of Fe_3_O_4_ NPs due to SDS coating during SDS@Fe_3_O_4_ NPs synthesis [36].

#### 3.1.2. Fourier Transform Infrared Spectroscopy (FTIR)

**Figure 2:**
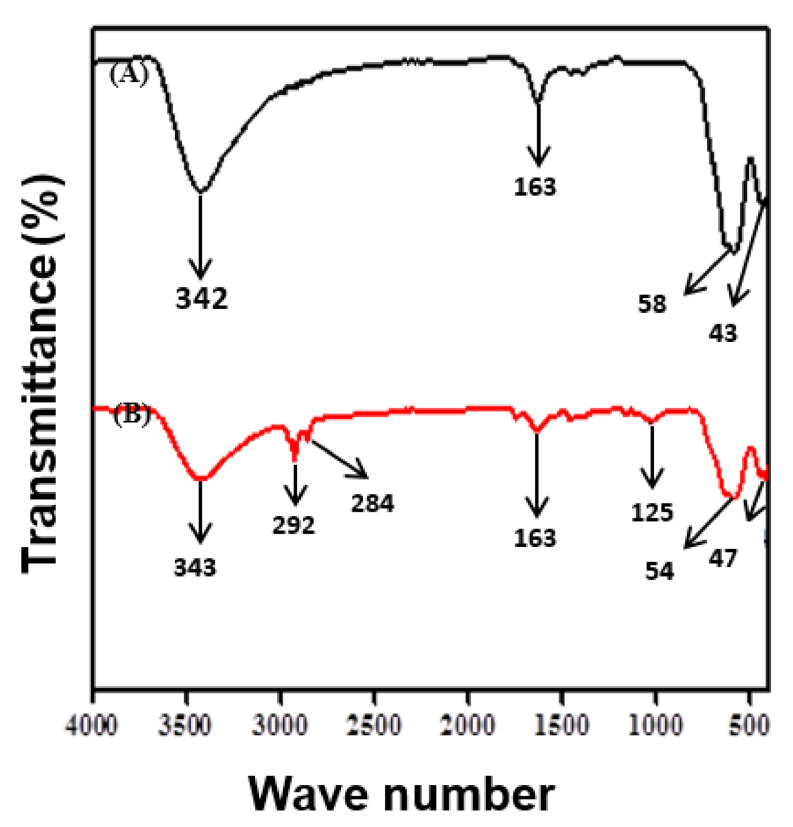
FTIR spectra of Fe_3_O_4_ (**A**) and SDS@Fe_3_O_4_ (**B**).

The FT-IR spectra of Fe_3_O_4_ and SDS@Fe_3_O_4_ NPs are shown in Figure 2. 

The two peaks at 585 and 435 cm^−1^, as shown in Figure 2A, correspond to the Fe-O bond vibrations of Fe_3_O_4_ NPs [37]. From these observations, it is confirmed the spinel structure of Fe_3_O_4_ NPs and also inferred the existence of the difference in the bond length in Fe-O. The peak at 3424 cm^−1^ in Figure 2A was associated to the O-H stretching vibrations arising from the hydroxyl group due to the presence of water molecules associated with Fe_3_O_4_ [38]. The H-O-H bending of water molecules in Figure 2A is observed at 1631 cm^−1^ in Fe_3_O_4_ NPs [39]. The FTIR spectrum of SDS@Fe_3_O_4_ NPs is shown in Figure 2B, which displayed a new absorption peak at 1252 cm^−1^ due to the stretching vibration of S=O groups of SDS and the presence of peaks at 2929 cm^−1^ and 2842 cm^−1^, which were assigned to the stretching mode for aliphatic C-H groups of SDS [40]. The peak at 1635 cm^−1^ in SDS@Fe_3_O_4_ (Figure 2B) was attributed to the H-O-H bending of water molecules and that at 3431 cm^−1^ was due to stretching vibration of hydroxyl group on the surface of the NPs. The presence of two peaks at 547 cm^−1^ and at 474 cm^−1^ in Figure 2B is attributed to Fe-O bonds in SDS-modified Fe_3_O_4_ [41]. Thus, the FTIR results confirmed successful synthesis of Fe_3_O_4_ NPs and their surface modifications through the adsorption of SDS molecules.

#### 3.1.3. Scanning Electron Microscopy (SEM)

**Figure 3:**
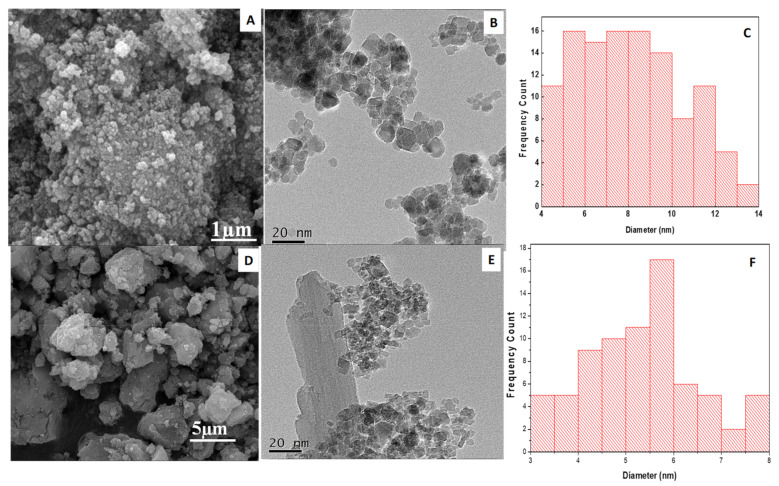
SEM image of Fe_3_O_4_ (**A**); TEM image of Fe_3_O_4_ (**B**); histogram images of Fe_3_O_4_ (**C**); SEM image of SDS@Fe_3_O_4_ (**D**); TEM image of SDS@Fe_3_O_4_ (**E**); histogram images of SDS@Fe_3_O_4_ (**F**). Note that scales of figures A and B are different.

The SEM micrograph of the synthesized magnetite (Fe_3_O_4_) NPs is shown in Figure 3A. It can be observed that the NPs exhibit spherical surface morphology, having a particle size lower than 100 nm scale with low polydispersity. The SEM image of the SDS@Fe_3_O_4_ NPs is shown in Figure 3D on the scale of up to 5 µm. The image depicts successful functionalization of Fe_3_O_4_ by SDS and the larger dispersion of SDS@Fe_3_O_4_ as compared with Fe_3_O_4_ NPs.

#### 3.1.4. Transmission Electron Microscopy (TEM)

The TEM micrograph of pristine Fe_3_O_4_ NPs (Figure 3B) on the scale of up to 20 µm shows their spherical shape with a narrow range particle size distribution centered at 9 ± 2 nm, as demonstrated by the histogram in Figure 3C. The TEM image of the SDS@Fe_3_O_4_ NPs is illustrated in Figure 3E. After coating with SDS, the size of SDS@Fe_3_O_4_ NPs appears to be smaller, as shown by the histogram (Figure 3F). This might be due to the coating of Fe_3_O_4_ NPs with SDS, which hinders NPs agglomeration. 

#### 3.1.5. Vibrating Sample Magnetometer (VSM)

**Figure 4:**
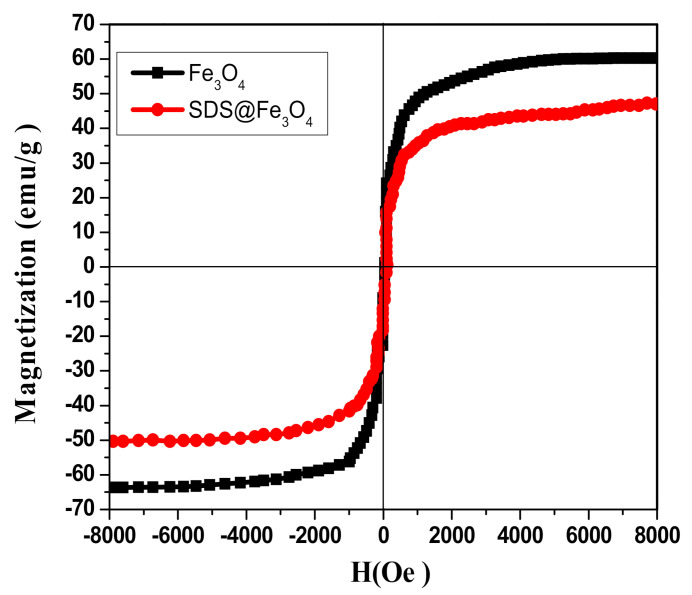
Magnetization curve for Fe_3_O_4_ and for SDS@Fe_3_O_4_ at room temperature.

The magnetic behavior of Fe_3_O_4_ and SDS@Fe_3_O_4_ NPs was studied by using VSM. As it may be seen in Figure 4, both Fe_3_O_4_ and SDS@Fe_3_O_4_ showed superparamagnetic behavior with different magnetic saturations level. The specific magnetic saturation magnitudes for Fe_3_O_4_ and SDS@Fe_3_O_4_ NPs were 60.0 and 50.0 emug^−1^, respectively, as displayed in Figure 4. Comparatively lower magnetic saturation of SDS@Fe_3_O_4_ NPs might be due to their coating with SDS [42]. In order to avoid aggregation of Fe_3_O_4_ NPs, which may severely reduce their catalytic efficiency, coating with SDS was executed in this work. In any case, as for the large magnetic saturation and superparamagnetic property of SDS@Fe_3_O_4_ NPs (Figure 4), such a coating did not affect the high efficiency in magnetic separation and recovery.

### 3.2. Degradation of RB by H_2_O_2_

**Figure 5:**
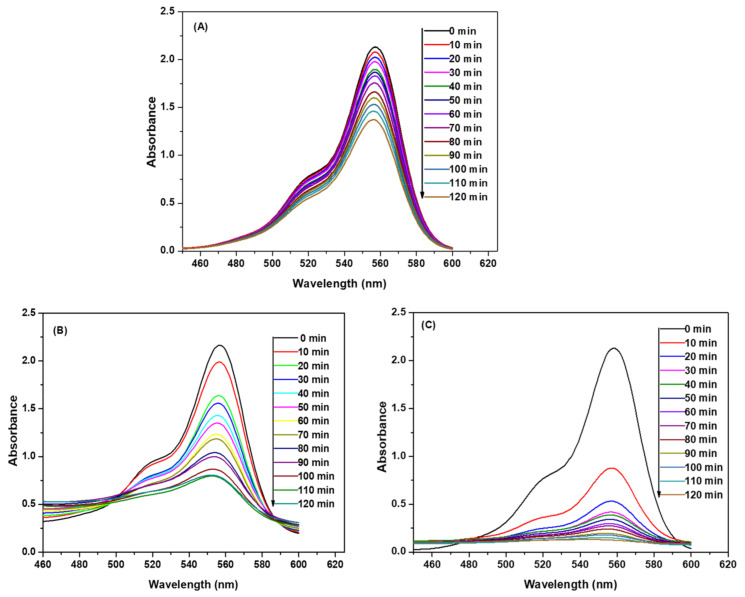
UV–visible spectra of Rhodamine B (RB) at various degradation times. In absence of Fe_3_O_4_ (**A**); in presence of Fe_3_O_4_ (**B**); and in presence of SDS@Fe_3_O_4_ (**C**). (Reaction conditions: 10 mg L^−1^ RB, 2.0 × 10^−1^ M H_2_O_2_, 0.1% *w*/*v* Fe_3_O_4_ NPs, 0.1% *w*/*v* SDS@Fe_3_O_4_, pH 3, and temperature 25 ± 2 °C).

The repetitive scans of the reactant mixture containing RB (10 mg L^−1^) and H_2_O_2_ (2 × 10^−1^ M) were recorded at constant time intervals of ten minutes in the visible region (460–600 nm). The temperature and pH were kept constant at 25 ± 0.2 °C and 3, respectively. These spectra, which are shown in Figure 5A, indicated that the absorbance intensities at λ_max_ (554 nm) progressively decreased with time. A decrease in the absorbance intensities was due to the degradation of RB by H_2_O_2_. 

The degradation of RB can be represented by the following representative reaction and rate Equation (1):

(1) $$\mathrm{RB}\;+\;H_{2}O_{2}\;\to \;\mathrm{Oxidized}\;\mathrm{Products}\;+\;H_{2}O,\;\mathrm{Rate}\;=\;\frac{-d\left[\mathrm{RB}\right]}{\mathrm{dt}}\;=\;k_{\mathrm{obs}}\left[\mathrm{RB}\right],$$

where $k_{\mathrm{obs}}$ is the observed value of the rate constant and was calculated from the slope of the plot of ln $\frac{{\left[\mathrm{RB}\right]}_{0}}{{\left[\mathrm{RB}\right]}_{t}}\;$versus t. The terms ${\left[\mathrm{RB}\right]}_{0}\;\mathrm{and}\;{\left[\mathrm{RB}\right]}_{t}$ are the concentrations of RB at time zero and at any time t, respectively. The observed rate constant depends upon the concentration of H_2_O_2_ as given by Equation (2). The order of the reaction is assumed to be x with respect to the concentration of H_2_O_2_.

(2) $${\;k}_{\mathrm{obs}}=k\;{\left[H_{2}O_{2}\right]}^{x}$$

where k is the specific rate constant with respect to H_2_O_2_ concentration. The values of k and x were respectively obtained from the intercept and slope of the plot of log${\;k}_{\mathrm{obs}}\;$versus log $\left[H_{2}O_{2}\right]$.

**Figure 6:**
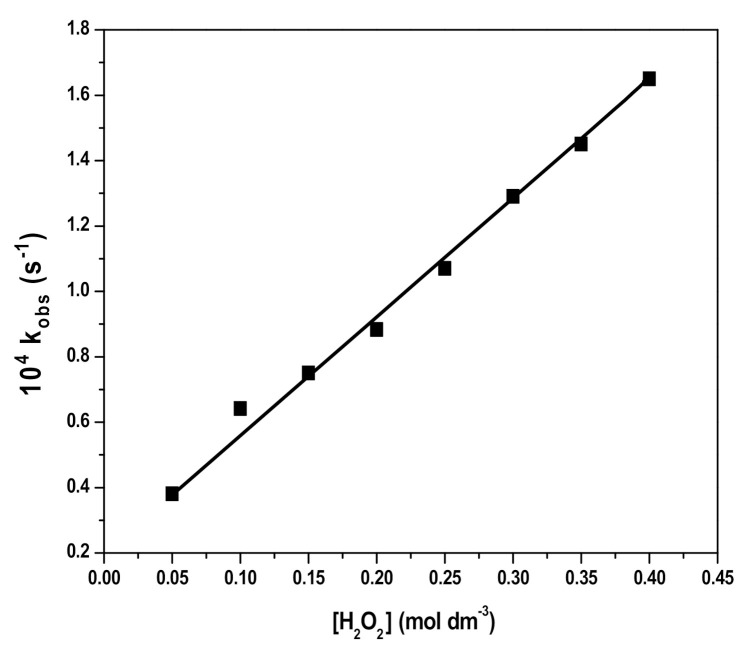
Plots of k_obs_ versus hydrogen peroxide (H_2_O_2_) concentration for the degradation of RB. (Reaction conditions: 10 mg L^−1^ RB, 5.0 × 10^−2^ to 4.0 × 10^−1^ M H_2_O_2_, pH 3, and temperature 25 ± 2 °C).

The rate of RB degradation was studied at varied concentrations of H_2_O_2_ in the range from 5.0 × 10^−2^ to 4.0 × 10^−1^ M while keeping a RB concentration of 10 mg L^−1^ at pH 3 and temperature 25 ± 0.2 °C. The values of rate constants were calculated and the plot of rate constant versus H_2_O_2_ concentration (Figure 6) shows a linear dependence of the rate constant values on H_2_O_2_ concentration. 

### 3.3. Degradation of RB in the Presence of Fe_3_O_4_ and SDS@Fe_3_O_4_ NPs

The addition of 0.1% *w*/*v* of Fe_3_O_4_ NPs to the solution containing RB and H_2_O_2_ increased the rate of degradation of RB, as is evident from the decrease in the rate of absorbance intensities with time, which is presented in Figure 5). The increase in the degradation rate of RB can be attributed to the catalytic role of Fe_3_O_4_ NPs. The degradation rate was further increased in the presence of 0.1% *w*/*v* SDS@Fe_3_O_4_ NPs as displayed in Figure 5C. 

**Figure 7:**
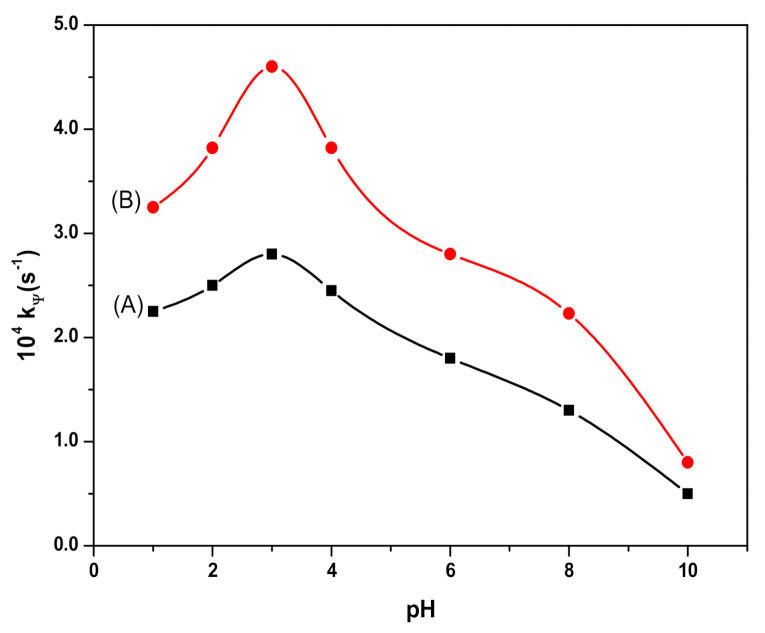
Effect of pH on the Rhodamine B (RB) degradation process. In presence of Fe_3_O_4_ (**A**); and in presence of SDS@Fe_3_O_4_ (**B**). (Reaction conditions: 10 mg L^−1^ RB, 2.0 × 10^−1^ M H_2_O_2_, 0.1% *w*/*v* Fe_3_O_4_, 0.1% *w*/*v* SDS@Fe_3_O_4_, and temperature 25 ± 2 °C).

In order to assess the effect of pH, the degradation rate of RB was studied in the pH range 1–10 by adjusting it with HCl/NaOH solutions. The observed results are presented in Figure 7. The plot of the rate constant versus pH (Figure 7) demonstrates that the values of the rate constant increase with pH until pH 3. Thereafter, on further increasing the pH beyond 3, the values of the rate constant decreased. Thus, a peaked behavior plot was obtained with the maximum degradation rate at pH 3.

**Figure 8:**
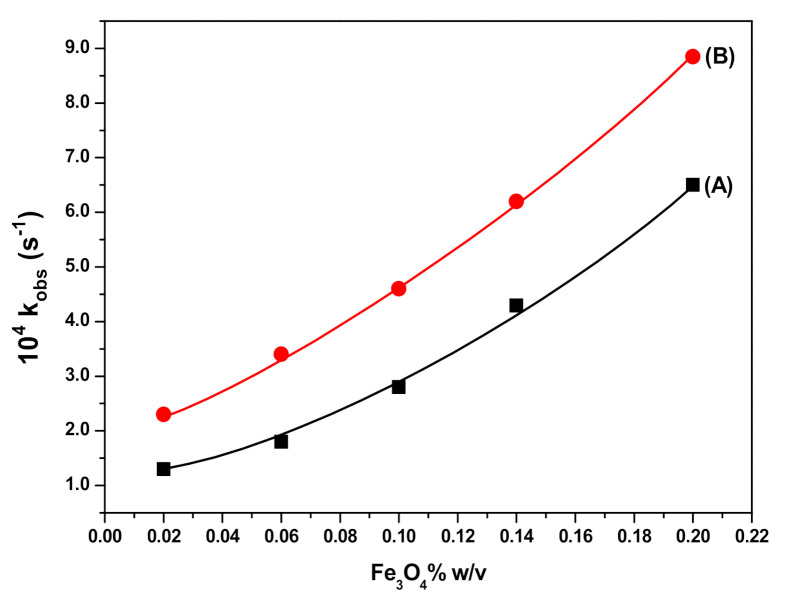
Plots of k_obs_ versus varying concentration of magnetic nanoparticles (NPs) for the degradation of the RB in presence of Fe_3_O_4_ (**A**), and in presence of SDS@Fe_3_O_4_ (**B**). (Reaction conditions: 10 mg L^−1^ RB, 2.0 × 10^−1^ M H_2_O_2_, 0.1% *w*/*v* Fe_3_O_4_, 0.1% *w*/*v* SDS@Fe_3_O_4_, pH 3, and temperature 25 ± 2 °C).

The influence of the magnetic Fe_3_O_4_ NPs dosage on the RB degradation rate was studied in the range between 0.02% and 0.2% *w*/*v* Fe_3_O_4_. The respective concentrations of H_2_O_2_ and RB were set at 2.0 × 10^−1^ M and 10 mg L^−1^, while the pH and temperature of the solution were 3 and 25 ± 0.2 °C, respectively. The increase in the amount of Fe_3_O_4_ increased the RB degradation rate, as shown by data graphically presented in Figure 8A. Furthermore, as it may be seen in Figure 8B, the influence of SDS@Fe_3_O_4_ concentration on the RB degradation rate showed the same pattern observed for Fe_3_O_4_, but with higher values of the rate constant. 

The observed enhancement in the rate of the RB degradation in presence of Fe_3_O_4_ can be described through the production of highly reactive hydroxyl radicals due to the interaction between the NPs and H_2_O_2_ [43,44], followed by the formation of peroxyl radicals and the subsequent oxidation of RB by these radicals, as described by the following reactions:

(i) ${\mathrm{Fe}}^{2+}+H_{2}O_{2}\to {\;\mathrm{Fe}}^{3+}\;+{\mathrm{HO}}^{*}+{\mathrm{OH}}^{-}$
  The possible reactions of free radicals are:
(ii) Fe^2+^$\;+{\;\mathrm{HO}}^{*}\to {\mathrm{Fe}}^{3+}+{\;\mathrm{OH}}^{-}$,
(iii) H_2_O_2_$\;+{\;\mathrm{HO}}^{*}\to$ H_2_O+ HO_2_^*^,
(iv) ${\mathrm{HO}}_{2}{}^{.}+{\mathrm{HO}}^{*}\to$ H_2_O + O_2_,
(v) ${\mathrm{HO}}^{*}+{\mathrm{HO}}^{*}\to$ H_2_O_2_,
(vi) ${\mathrm{HO}}^{*}$ + RB → Products,
(vii) ${\mathrm{HO}}_{2}^{*}$ + RB → Products.

**Figure 9:**
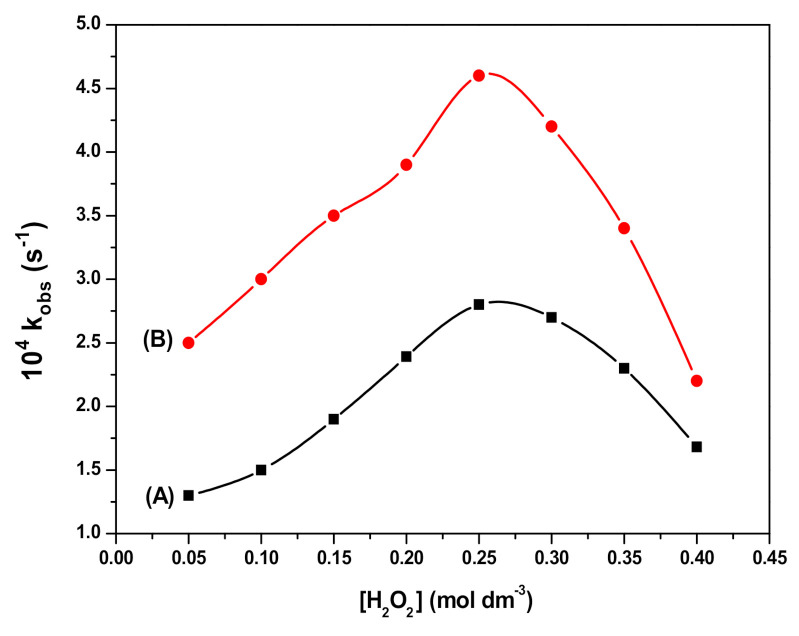
Effect of H_2_O_2_ concentration on RB degradation. In presence of Fe_3_O_4_ (**A**); and in presence of SDS@Fe_3_O_4_ **(B**). (Reaction conditions: 10 mg L^−1^ RB, 5.0 × 10^−2^ to 4.0 × 10^−1^ M H_2_O_2_, 0.1% *w*/*v* Fe_3_O_4_, 0.1% *w*/*v* SDS@Fe_3_O_4_, pH 3, and temperature 25 ± 2 °C).

Thus, the oxidation of RB by HO*and ${\mathrm{HO}}_{2}{}^{*}$ radicals leads to a decrease in its concentration. As for the RB degradation in the presence and absence of Fe_3_O_4_ and SDS@Fe_3_O_4_, results shown in Figure 5 to 8 allow to state that RB degradation was enlarged under the presence of Fe_3_O_4_ and SDS@Fe_3_O_4_ NPs. As for the degradation rate of RB, it was linearly dependent on the initial concentration of H_2_O_2_ in the absence of NPs. The RB degradation was comparatively higher in presence of SDS@Fe_3_O_4_ than Fe_3_O_4_ and increased with the increase in the dosage of either Fe_3_O_4_ or SDS@Fe_3_O_4_ NPs. The variations in pH displayed a similar influence on the RB degradation rate in the presence of Fe_3_O_4_ and SDS@Fe_3_O_4_ NPs, the rate showing a peaked behavior. From these observations, it was confirmed that the reaction proceeded through the formation of highly reactive free radicals in the presence of Fe_3_O_4_ and SDS@Fe_3_O_4_ NPs due to the interaction between Fe^2+^ and H_2_O_2_, as described by reactions (i) to (vii). The increase in the amount of the NPs increases the production of ${\mathrm{HO}}^{*}$radicals (step (i)) and, therefore, an enhancement in the RB degradation rate was observed with the increase in the NPs dosage, which is coincident with previous observations [45]. As shown in Figure 6, the RB degradation rate increased from 0.4 to 1.7 × 10^4^ s^−1^ with increasing H_2_O_2_ concentration in the absence of NPs. In Figure 9, under the presence of NPs, larger degradation rates are represented, varying between 1.3 and 2.8 × 10^4^ s^−1^ in the case of Fe_3_O_4_ and between 2.6 and 4.8 × 10^4^ s^−1^ in the case of SDS@Fe_3_O_4_, which compare rather well with published rate constants for the catalytic degradation of RB (Table S1 in the Supplementary Materials). However, as it may be seen in Figure 9, in the presence of Fe_3_O_4_ and SDS@Fe_3_O_4_ NPs, the rate constant value increased with the concentration of H_2_O_2_ until it was 2.5 × 10^−1^ M H_2_O_2_, and thereafter decreased with H_2_O_2_ concentration. After the maximum, this decreasing effect in the rate constant with the increase in the H_2_O_2_ concentration was due to the other free radical reactions taking place in steps (ii) to (v). Thus, at higher concentrations of H_2_O_2_, the side reactions scavenged the ${\mathrm{HO}}^{*}$ radicals and decreased the concentration of free radicals available to oxidize the dye and, therefore, the rate of the reaction decreased [46].

The rate of degradation of RB was highly pH-dependent and, as it is shown in Figure 7, the maximum rate of degradation was observed at pH 3 in the presence and the absence of NPs. At high concentrations of H^+^ ions (pH < 3), peroxide gets solvated to form stable oxonium ions, which enhanced the activity of H_2_O_2_ and restricted the generation of hydroxyl radicals [47,48,49]. Moreover, the excess of H^+^ ions acts as hydroxyl radical scavenger and, with the increase in H^+^ ions, the concentration of ${\mathrm{HO}}^{*}$ radicals decreases, thus, decreasing the rate of reaction [48]. Furthermore, the strong electrostatic interaction between the anionic surfactant head groups and cationic dye molecules at lower pH also decreases the rate of RB degradation. The observed lower rate of reaction at higher pH may be related to the formation of the Fe^3+^-complexes, which decreases the dissolved Fe^2+^ ions that were available to generate free radicals [49]. 

The higher degradation rate of RB in the presence of SDS@Fe_3_O_4_ NPs in comparison with bare Fe_3_O_4_ NPs that is observed in Figure 8, might be due to the larger capture of RB by SDS@Fe_3_O_4_ than by Fe_3_O_4_. Thus, the generated free radicals at the NPs surface can readily attack the attached RB and thus leading to the increase in RB degradation rate. Binding of RB to the SDS@Fe_3_O_4_ surface can be explained by the electrostatic interaction between the anionic surfactant and protonated cationic dye at pH 3 [50].

### 3.4. Effect of SDS Concentration and Fe_3_O_4_ NPs Dosage on RB Degradation 

**Figure 10:**
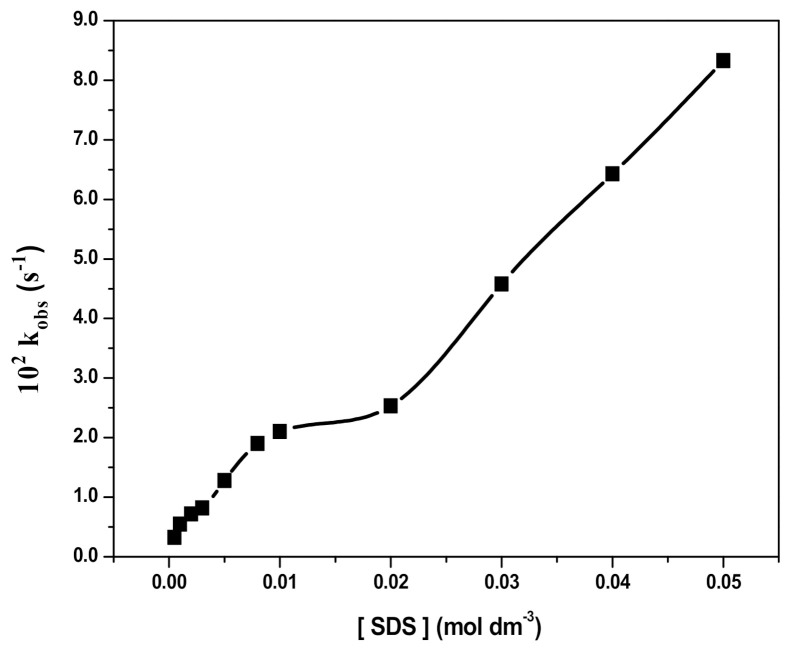
Effect of sodium dodecyl sulfate (SDS) concentration on RB degradation. (Reaction conditions: 10 mg L^−1^ RB, 2.0 × 10^−1^ M H_2_O_2_, 0.1% *w*/*v* Fe_3_O_4_, pH 3, and temperature 25 ± 2 °C).

**Figure 11:**
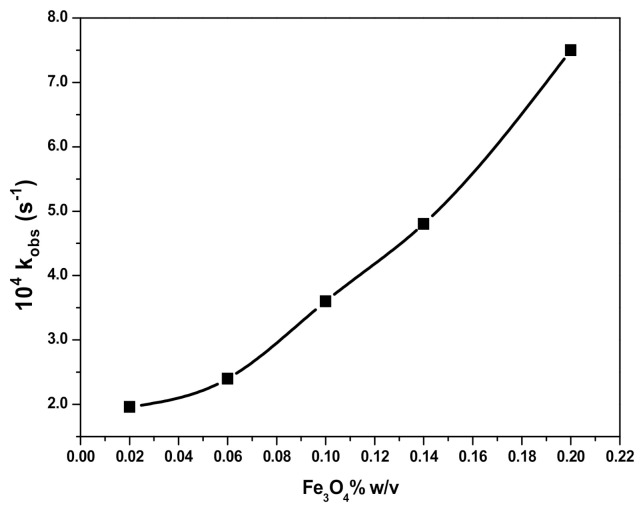
Effect of Fe_3_O_4_ concentration on RB degradation. (Reaction conditions: 10 mg L^−1^ RB, 0.02% to 0.2% *w*/*v* Fe_3_O_4_, 2.0 × 10^−2^ M SDS, pH 3, and temperature 25 ± 2 °C).

The addition of SDS at varied concentrations (5.0 × 10^-4^ to 5.0 × 10^−2^ M) to a solution containing RB (10 mgL^−1^), H_2_O_2_ (2.0 × 10^−1^ M), and Fe_3_O_4_ (0.1% *w*/*v*) NPs at pH 3 resulted in an increase in the rate of the degradation reaction, as shown in Figure 10. On the other hand, an increase in the amount of Fe_3_O_4_ NPs from 0.02% to 0.2% *w*/*v* at a fixed concentration of SDS (2.0 × 10^−2^ M) also increased the rate of RB degradation, as shown in Figure 11. 

The respective degradation rates of RB in presence of SDS and Fe_3_O_4_ can be represented by Equations (3) and (4).

(3) $$\mathrm{RB}\;+\;D_{n}\;\overset{K_{s}}{\Leftrightarrow }\;{\mathrm{RB}}_{\mathrm{mic}}\;\overset{H_{2}O_{2}}{\to }\;\mathrm{Products},$$

(4) $${\mathrm{Fe}}_{3}O_{4}\;+\;{\mathrm{RB}}_{\mathrm{mic}}\;\overset{K_{e}}{\Leftrightarrow }\;{\mathrm{RB}}_{\mathrm{mic}}-{\mathrm{Fe}}_{3}O_{4}\;\overset{H_{2}O_{2}}{\to }\;\mathrm{Products}.$$

The presence of SDS micelles (D_n_) partitions RB into micellar (RB_mic_) and aqueous pseudo-phases resulting into the retardation of RB oxidation with H_2_O_2_ in the presence of SDS, which may be related to the electrostatic repulsion and, therefore, separation between the species involved in the reaction. However, in the presence of Fe_3_O_4_, micellised RB (RB_mic_) is incorporated to the NPs surface to form RB_mic_−Fe_3_O_4_ where H_2_O_2_ interacts to form reactive ${\mathrm{HO}}^{*}$radicals readily available to oxidize RB at the same site. Therefore, RB degradation is catalyzed and the rate of the reaction increases with increasing SDS concentration in the presence of Fe_3_O_4_ NPs (Figure 10) and also with increasing the amount of Fe_3_O_4_ NPs in the presence of SDS (Figure 11). In Figure 10, a two steps increase of the degradation may be observed, which may be related to the formation of premicellar aggregates below the critical micelle concentration (cmc) of SDS and micelles above cmc [8,10,51,52], then increasing micelles formation with SDS concentration. Regarding Figure 11, at a SDS concentration above cmc, an increasing degradation rate occurred under increasing Fe_3_O_4_ concentration, as previously observed in Figure 8 and explained by reactions (i) to (vii). These results are in agreement with previous studies on RB photocatalytic degradation [53,54]. 

### 3.5. Effect of Temperature on RB Degradation 

The effect of temperature on RB degradation (10 mg L^−1^) in aqueous solutions in the presence of H_2_O_2_ (2.5 × 10^−1^ M) and at pH 3 was studied at varied temperatures ranging from 25 to 60 °C (because above 60 °C, due to thermal disintegration of H_2_O_2_ and free radicals, the rate of RB degradation slowed down) in the absence or in the presence of Fe_3_O_4_ NPs (0.1% *w*/*v*), Fe_3_O_4_ NPs (0.1% *w*/*v*) together with SDS (2.0 × 10^−2^ M) or SDS@Fe_3_O_4_ (0.1% *w*/*v*). 

The energy of activation was calculated using the Arrhenius equation Equation (5), which gave a straight line plot for log k versus 1/T.

(5) $$\log k_{\mathrm{obs}}=-\frac{E_{a}}{2.303\;\mathrm{RT}}+\log A_{o}$$

where E_a_ is the activation energy (kJ mol^−1^), R (8.314 J mol^−1^K^−1^) is the universal gas constant, T is the temperature in Kelvin (K), A_o_ is the frequency factor, and k_obs_ is the measured first-order rate constant. The E_a_ was determined from the slope and values are given in Table 1.

The value of ΔH (enthalpy of activation) and ΔS (entropy of activation) were calculated using the Erying equation Equation (6).

(6) $$\ln\left(\frac{k_{\mathrm{obs}}}{T}\right)=-\frac{∆H}{R}\times \frac{1}{T}+\ln\frac{k_{B}}{h}+\frac{∆S}{R},$$

where $k_{B}$ is the Bolzmann’s constant and h is the Plank’s constant. A plot of ln (k_obs_/T) versus 1/T produces a straight line and the values of ΔH and ΔS may be obtained from the slope and the intercept, respectively. The so determined ΔH and ΔS values are given in Table 1. 

As it may be seen in Table 1, good fittings (R^2^ > 0.94) to the Erying equation were obtained within the temperature range here considered. The largest E_a_ and ΔH determined for RB degradation were those in the absence of NPs. These values progressively decreased in the presence of Fe_3_O_4_ NPs, Fe_3_O_4_ NPs together with SDS and SDS@Fe_3_O_4_, which provided the lowest E_a_ and ΔH. Regarding the ΔS, although the effect was not so remarkable as for E_a_ and ΔH, slightly lower values were also determined under the presence of NPs. These results point to the energetically favorable effect of Fe_3_O_4_ and SDS@Fe_3_O_4_ NPs, which confirms that these are efficient catalysts.

## 4. Conclusions

In this work, Fe_3_O_4_ NPs were synthesized, coated with SDS to synthesize SDS@Fe_3_O_4_ NPs, and both tested as catalysts for the oxidation of RB under H_2_O_2_. The main novelty was to compare the dye degradation under three different situations, namely, in presence of just H_2_O_2_, of H_2_O_2_ and Fe_3_O_4_ NPs, and H_2_O_2_ and SDS@Fe_3_O_4_ NPs. Observed pseudo-first-order kinetic rates (k_obs_, s^−1^) for the degradation of RB (10 mg L^−1^) at pH 3 and temperature 25 ± 2 °C were between 0.4 and 1.7 × 10^4^ s^−1^, linearly dependent upon H_2_O_2_ concentrations within 5.0 × 10^−2^ to 4.0 × 10^−1^ M. Under identical experimental conditions, except for the presence of 0.1% *w*/*v* NPs, the observed rates increased to values between 1.3 and 2.8 × 10^4^ s^−1^ in the case of Fe_3_O_4_ and between 2.6 and 4.8 × 10^4^ s^−1^ in the case of SDS@Fe_3_O_4_. Fe_3_O_4_ NPs with H_2_O_2_ gave readily the highly reactive hydroxyl radicals, which enhanced the rate of RB degradation. Furthermore, an increased catalytic effect was observed for SDS@Fe_3_O_4_ because the SDS coating avoided Fe_3_O_4_ aggregation and the consequent efficiency depletion. However, under the presence of Fe_3_O_4_ and SDS@Fe_3_O_4_ NPs, k_obs_ did not increase linearly with H_2_O_2_ concentration but just until 2.5 × 10^−1^ M H_2_O_2_, then decreased with increasing H_2_O_2_ concentration, which was associated to free radical competitive reactions. On the other hand, it was verified that the addition of SDS molecules to the dye solution containing Fe_3_O_4_ also increased the rate of reaction, which was related to the incorporation of micellized RB ions onto the Fe_3_O_4_ NPs surface. Overall, this work demonstrated that the application of Fe_3_O_4_ and SDS@Fe_3_O_4_ along with H_2_O_2_ can be an efficient method for the rapid removal of cationic dyes from wastewater in line with the green chemistry principles.

## Figures and Tables

**Table 1:** Activation parameters determined for RB degradation by H_2_O_2_ in the absence and presence of Fe_3_O_4_ and SDS and SDS@Fe_3_O_4_.

Reaction Media	E_a_ (kJ mol^−1^)	ΔH (kJ mol^−1^)	ΔS (J mol^−1^K^−1^)	R^2^
H_2_O_2_	69.47	66.85	–132.66	0.949
H_2_O_2_ + Fe_3_O_4_	28.47	25.86	–188.18	0.953
H_2_O_2_ + Fe_3_O_4_ + SDS	32.47	29.86	–194.42	0.945
H_2_O_2_ + SDS@Fe_3_O_4_	15.63	13.01	–149.00	0.953

Reaction conditions: 10 mg L^−1^ RB, 2.0 × 10^−1^ M H_2_O_2_, 0.1% *w*/*v* Fe_3_O_4_ (when present), 0.1% *w*/*v* SDS@Fe_3_O_4_ (when present), 2.0 × 10^−2^ M SDS, pH 3, and varied temperatures (between 25 and 60 ± 2 °C).

## Supplementary Materials

The following are available online at https://www.mdpi.com/2073-4360/12/10/2246/s1, Table S1: Published results on the rate constant (k_obs_, s^−1^) for the catalytic degradation of RB using different catalysts.
